# Editorial: Systems Biology and Bioinformatics in Gastroenterology and Hepatology

**DOI:** 10.3389/fphys.2019.01438

**Published:** 2019-11-22

**Authors:** Peter L. M. Jansen, Kai Breuhahn, Andreas Teufel, Steven Dooley

**Affiliations:** ^1^Emeritus Professor of Hepatology, Amsterdam University Medical Center, Amsterdam, Netherlands; ^2^Institute of Pathology, University Hospital Heidelberg, Heidelberg, Germany; ^3^Division of Hepatology, Division of Clinical Bioinformatics, Department of Medicine II, Medical Faculty Mannheim, Heidelberg University, Heidelberg, Germany; ^4^Division of Molecular Hepatology, Department of Medicine II, Medical Faculty Mannheim, Heidelberg University, Heidelberg, Germany

**Keywords:** gastroenterology, hepatology, systems biology, systems medicine, mathematical modeling

## How Systems Medicine Improves Our Understanding of Complex Gastroenterological Diseases

Traditional medical research gained tremendous improvements in detection and treatment of acute and chronic metabolic and inflammatory diseases as well as cancer. Especially the field of hepatology and gastroenterology has significantly benefitted from these advances. Indeed, the discovery of basic molecular and cellular disease mechanisms in the last 60 years led to the development of reliable diagnostic tests and effective therapies. For instance, the discovery of the hepatitis B and C viruses (HBV, HCV) led to powerful diagnostic tools, antiviral drugs, and an HBV vaccine (Szmuness et al., 1980, 1981; André, 1990; Lau and Wright, 1993). Indeed, mass vaccination in Taiwan led to a significant reduction of HBV prevalence and hepatocellular carcinoma incidence (Chang et al., 1997, 2016). Furthermore, the development of direct-acting antiviral drugs allows the eradication of HCV (Das and Pandya, 2018). These achievements occurred in a relatively short period of time. For example, HCV was discovered in 1989 and the first effective antiviral therapy for one genotype was developed only 20 years later (Boettler et al., 2019; Viganò et al., 2019; Zajac et al., 2019). Equally, for many monogenetic liver diseases reliable diagnostic tests exist (Lammert, 2016; Weber and Lammert, 2017). Although effective pharmacotherapy for some of these diseases exists (Wilson's disease), comparable treatments are not available for others (e.g., progressive familial intrahepatic cholestasis, PFIC). In this context, translation of gene knock-out mouse models emerged as powerful tool to understand the underlying disease processes (Liu, 2013) and may eventually lead to successful gene therapy.

In contrast to mono-factorial diseases caused by viruses or individual gene alterations, the situation is quite different in more complex multi-factorial diseases. For example non-alcoholic fatty liver disease (NAFLD) was first described by the pathologist Jurgen Ludwig in 1980 (Ludwig et al., 1980) but no effective therapy is currently available, 40 years later (Altinbas et al., 2015; Gottlieb et al., 2019). Despite all recent success in the development of treatments of HBV and HCV, targeting multi-factorial metabolic liver disease like NAFLD or the more serious non-alcoholic steatohepatitis (NASH) is still difficult. It remains unclear if targeting liver disease alone will pave the way to success or if broader approaches will ultimately lead to a decrease in patient's mortality and morbidity. Importantly, a plethora of reasons significantly complicates the systematic analysis of complex diseases:

1. Not single genes, but the identification of gene signatures would probably improve our understanding of NAFLD/NASH development and progression. This complex molecular behavior is usually affected by cellular processes, as well as paracellular communication networks (Gottlieb et al., 2019).
2. The dynamic and/or spatial organization of molecular and biochemical processes is controlled by overarching superior mechanisms (e.g., circadian rhythm) or by gender differences (Hashimoto and Tokushige, 2011; Pan and Fallon, 2014).
3. Cellular signaling pathways form dense interacting networks and it is difficult to predict how changes in one parameter/pathway affect other parameters/pathways under distinct conditions (Teufel et al., 2016; Bessone et al., 2019; Pierantonelli and Svegliati-Baroni, 2019).
4. Complex diseases such as NAFLD/NASH are multi-organ diseases affected by the brain, gut, the gut micro-flora, pancreas, subcutaneous, and abdominal fat (Konturek et al., 2011; Borrelli et al., 2018; Kolodziejczyk et al., 2019; Milosevic et al., 2019).

The multi-scale and multi-stage complexity of NAFLD and NASH, and the necessity to perform one or more liver biopsies to stage and monitor the disease, form a considerable obstacle in the development and application of targeted precision medicine. For instance, the anti-oxidant vitamin E is a recommended therapy in non-diabetic patients with biopsy-proven NASH (Sanyal et al., 2010; Chalasani et al., 2018). In real life only a minority of patients receives this drug (Ratziu et al., 2012). The FXR-agonist obeticholic acid also showed positive results in biopsy-proven non-cirrhotic patients with NASH (Neuschwander-Tetri et al., 2015). The mechanism of action of this drug in NAFLD is unclear and long-term effects and safety need to be assessed. Treatment of cirrhotic patients with this drug cannot be recommended. For successful wide-scale pharmacotherapy programs, non-invasive disease biomarkers are clearly needed.

NAFLD represents a multi-scale disease, in which the entire metabolic program of liver hepatocytes (incl. structures at the molecular level and subcellular organelles), non-parenchymal cells (incl. cholangiocytes, endothelial cells, Kupffer cells, and hepatic stellate cells), and the blood stream (incl. the presence of immune cells and sub-cellular blood components) are critically involved in disease development and progression. In addition, there is dysfunctional temporal communication between organs such as liver, gut, brain, pancreas and fatty tissues. Indeed, NAFLD develops in 20–30 years, starting from “simple” steatosis and progresses to pronounced liver cirrhosis. These dynamic spatial and temporal changes significantly increase the level of complexity and further complicate biomarker and drug development. For instance, pharmacotherapy targeting steatosis may be more effective in early disease stages while drugs that act on inflammation and fibrosis are more suitable at later stages. Once advanced cirrhosis with profound architectural changes of the liver and portal hypertension is established, effective pharmacotherapy becomes even more difficult.

Are computational approaches and systems medicine the solution for complex diseases? Dynamic processes can be described mathematically with a set of differential equations. With a number of these equations, scientists can generate computational models, which can describe the time-resolved behavior of molecular reactions and cellular processes (Schliess et al., 2014; Meyer et al., 2017; Berndt et al., 2018; Hoehme et al., 2018; Lucarelli et al., 2018; Poloznikov et al., 2018; Kockerling et al., 2019). In this process, experimentalists provide quantitative and semi-quantitative data derived from *in vitro* and *in vivo* models to feed these mathematical constructs. Once a reliable and robust computational model is established, the model can be used for *in silico* research, an approach that has the potential to save laboratory animals and to protect people and patients before a drug is used or tested in a clinical setup.

This model-based gain of knowledge leads to a process of iteration and re-iteration between theoretical and experimental scientists until the mathematical model is a reliable proxy of the *in vivo* situation. Sometimes predictions cannot perfectly reflect the processes observed in living cells or organisms; however, these complications can also lead to new scientific knowledge. One example within one of the biggest systems biology consortia (LiSyM, see below) was the finding that ammonia detoxification was less affected by damaging the centri-zonal glutamine synthase-containing hepatocytes than predicted. These unexpected findings led to the discovery of a novel ammonia detoxification pathway (Schliess et al., 2014).

Since 15 years the German Ministry of Education and Research (BMBF) fosters systems biology and systems medicine by supporting the collaboration of multidisciplinary research groups, including biologists, clinical researchers, and mathematicians, working on liver physiology and liver diseases, including NAFLD. The research network HepatoSys was launched in 2004 to study the processes in liver cells with a systems biology approach. It was Europe's first funding measure in this field. The follow-up project the “Virtual Liver Network (VLN)” took the systems biology liver research to the next biological level. Drawing on the findings at cellular level, the network examined the processes for the whole organ. The initiative was the first systems biology network that focused on an entire organ. The current funding activity “Research Network Systems Medicine of the Liver—LiSyM” builds again on the results produced by HepatoSys and VLN. LiSyM aims to transfer the computational models into clinical application for use as diagnostic tools to assist doctors in choosing the most appropriate therapy. LiSyM and the proceeding initiatives have been successful over the years in developing computational models that help theoreticians and experimentalists to discover new aspects of signaling pathways and mechanisms to test the therapeutic potential of new molecules or biological agents *in vivo* as *in silico* (www.lisym.org).

However, opportunities to discuss the diverse facets of systems biology from data generation, utilization of mathematical models, and data integration among experts in the field remain rare. In this regard, we were happy that Frontiers in Physiology provided a platform for such urgently needed discussions and visibility beyond the German networks. The collection of articles in this special issue of Frontiers in Physiology provides examples of the current status of research in gastrointestinal diseases, including NAFLD, alcoholic hepatitis, viral hepatitis, liver fibrosis, and liver cancer, applying systems biology at the level of cells, zones, tissues, networks, and with regard to systemic consequences.

The issue comprises 20 articles from more than 170 authors. From June 2017, where the first article was accepted, until July 30 2019, the manuscripts of the issue have nearly 46,000 views. In more detail, 1 review and 19 original articles are included with nearly half of it (9 in total) from participants of the BMBF-funded networks VLN and LiSyM.

Twelve contributions are related to liver, 3 to colon and gastrointestinal tract and 1 to pancreas, again highlighting the predominant role of the German network in this new scientific field. Experimental data of the contributions include gene expression arrays (4), metabolomics data (6), proteomics data (3), imaging (2), and signal transduction pathways (6). The modeling type of the manuscripts include high throughput data and bioinformatics in 10 cases and mathematical modeling in 9 contributions. We believe that this initiative successfully provided a platform for researchers and clinicians who are interested in systems medicine with focus on gastroenterology and hepatology.

## Author Contributions

All authors listed have made a substantial, direct and intellectual contribution to the work, and approved it for publication.

### Conflict of Interest

The authors declare that the research was conducted in the absence of any commercial or financial relationships that could be construed as a potential conflict of interest.
